# The Natural Stilbenoid (–)-Hopeaphenol Inhibits HIV Transcription by Targeting Both PKC and NF-κB Signaling and Cyclin-Dependent Kinase 9

**DOI:** 10.1128/aac.01600-22

**Published:** 2023-03-15

**Authors:** Ian Tietjen, Cole Schonhofer, Amanda Sciorillo, Maya E. Naidu, Zahra Haq, Toshitha Kannan, Andrew V. Kossenkov, Jocelyn Rivera-Ortiz, Karam Mounzer, Colin Hart, Kwasi Gyampoh, Zhe Yuan, Karren D. Beattie, Topul Rali, Kristy Shuda McGuire, Rohan A. Davis, Luis J. Montaner

**Affiliations:** a The Wistar Institute, Philadelphia, Pennsylvania, USA; b Faculty of Health Sciences, Simon Fraser University, Burnaby, British Columbia, Canada; c Jonathan Lax Immune Disorders Treatment Center, Philadelphia Field Initiating Group for HIV-1 Trials, Philadelphia, Pennsylvania, USA; d Griffith Institute for Drug Discovery, School of Environment and Science, Griffith University, Brisbane, Queensland, Australia; e School of Natural and Physical Sciences, The University of Papua New Guinea, Port Moresby, Papua New Guinea

**Keywords:** antiviral pharmacology, cyclin kinase inhibitors, human immunodeficiency virus, natural antimicrobial products, transcriptional regulation, viral latency, viral pathogenesis

## Abstract

Despite effective combination antiretroviral therapy (cART), people living with HIV (PLWH) continue to harbor replication-competent and transcriptionally active virus in infected cells, which in turn can lead to ongoing viral antigen production, chronic inflammation, and increased risk of age-related comorbidities. To identify new agents that may inhibit postintegration HIV beyond cART, we screened a library of 512 pure compounds derived from natural products and identified (–)-hopeaphenol as an inhibitor of HIV postintegration transcription at low to submicromolar concentrations without cytotoxicity. Using a combination of global RNA sequencing, plasmid-based reporter assays, and enzyme activity studies, we document that hopeaphenol inhibits protein kinase C (PKC)- and downstream NF-κB-dependent HIV transcription as well as a subset of PKC-dependent T-cell activation markers, including interleukin-2 (IL-2) cytokine and CD25 and HLA-DRB1 RNA production. In contrast, it does not substantially inhibit the early PKC-mediated T-cell activation marker CD69 production of IL-6 or NF-κB signaling induced by tumor necrosis factor alpha (TNF-α). We further show that hopeaphenol can inhibit cyclin-dependent kinase 9 (CDK9) enzymatic activity required for HIV transcription. Finally, it inhibits HIV replication in peripheral blood mononuclear cells (PBMCs) infected *in vitro* and dampens viral reactivation in CD4^+^ cells from PLWH. Our study identifies hopeaphenol as a novel inhibitor that targets a subset of PKC-mediated T-cell activation pathways in addition to CDK9 to block HIV expression. Hopeaphenol-based therapies could complement current antiretroviral therapy otherwise not targeting cell-associated HIV RNA and residual antigen production in PLWH.

## INTRODUCTION

While combination antiretroviral therapy (cART) effectively blocks viral replication and has significantly reduced mortality due to HIV/AIDS, no approved therapies to date directly target HIV transcription in CD4^+^ T cells containing integrated and transcriptionally competent provirus. As a result, people living with HIV (PLWH) sustain levels of cell-associated viral RNA and/or viral antigen in circulating CD4^+^ T cells with higher expression in organs such as the gut (1, 2). In virally suppressed PLWH on long-term ART, ongoing persistence of HIV is associated with higher immune activation, which in turn has been linked to increased risk of cancers, cardiovascular disease, and other age-related comorbidities (3–6). Chemical compounds that can block viral transcription from infected CD4^+^ T cells on cART would be expected to lower viral antigen production and its potential to contribute to immune activation.

Viral transcription is regulated at the HIV long-terminal repeat (LTR) by the viral Tat protein (7). In the absence of Tat, RNA polymerase II-mediated transcription from the LTR stalls at the RNA transactivation response (TAR) element stem-loop structure. To enhance viral transcription, Tat binds the host positive transcription elongation factor B (P-TEFb) complex, consisting of cyclin-dependent kinase 9 (CDK9) and cyclin T1, and recruits P-TEFb to the TAR element. Once recruited, CDK9 phosphorylation of RNA polymerase II results in productive HIV transcription (8–10). Host factors, including NF-κB, which is induced by both protein kinase C (PKC) activation and tumor necrosis factor alpha (TNF-α) signaling, can further enhance LTR-mediated HIV transcription (7, 11, 12).

We previously screened a small marine natural product library and identified bengamide A as a potent inhibitor of NF-κB signaling and postintegration virus production (13). To discover additional chemical leads that can inhibit HIV transcription, here we used a chronically HIV-infected cell line to screen an additional 512 pure compounds from the Davis open access natural product-based library, which contains pure natural products and natural product derivatives obtained from plants, mushrooms, and marine invertebrates of Australia, Papua New Guinea, and elsewhere (14, 15). From this screen, we identified (–)-hopeaphenol, a plant-derived stilbenoid, as a selective inhibitor of HIV transcription that targets in part PKC- and NF-κB-mediated HIV transcription and CDK9 activity in T cells, resulting in the inhibition of virus production *in vitro* and infectious virus replication in peripheral blood mononuclear cells (PBMCs).

## RESULTS

### Identification of (–)-hopeaphenol as an inhibitor of HIV expression.

**FIG 1:**
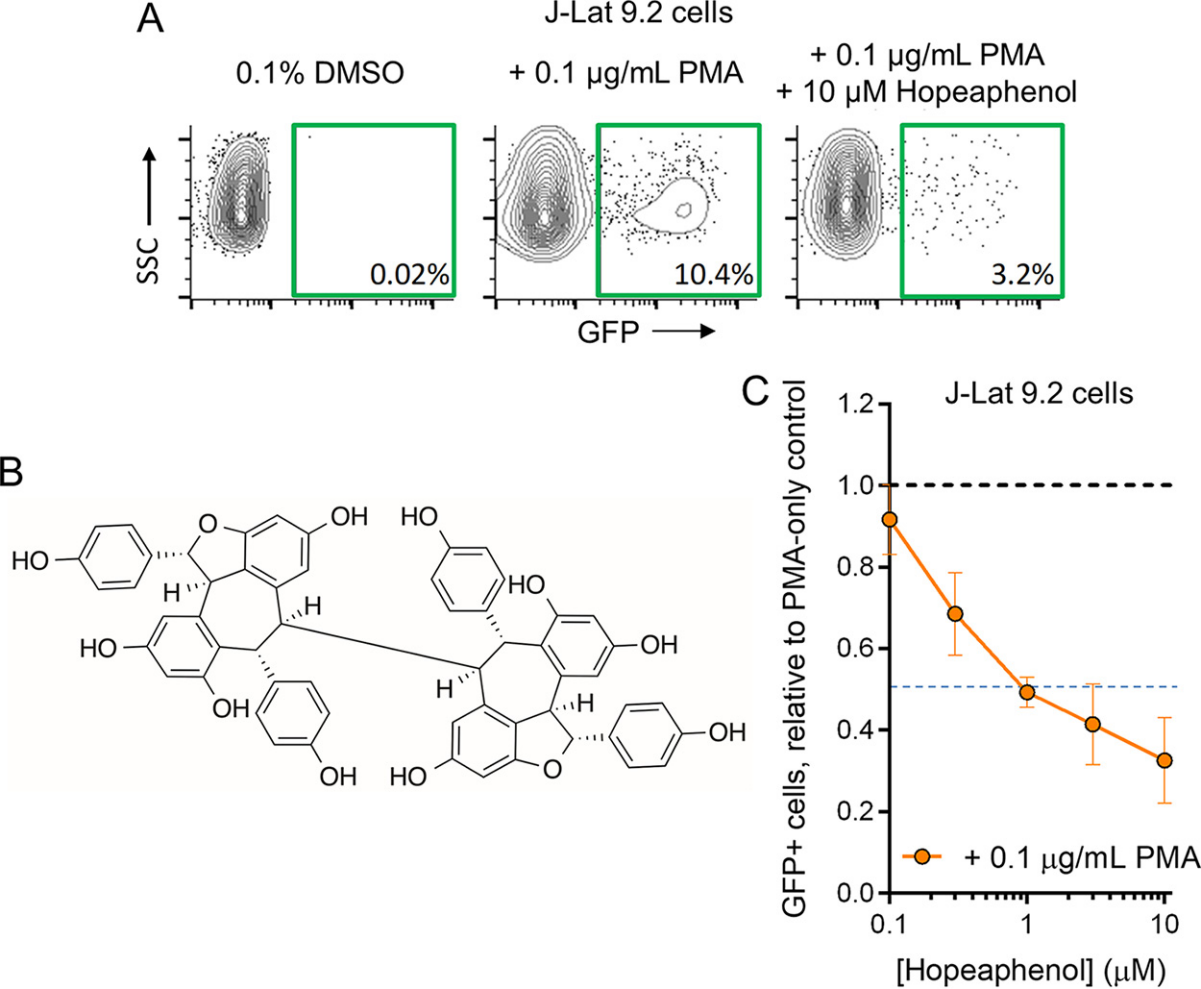
Overview of HIV inhibitor screening strategy and identification of (–)-hopeaphenol. (A) Representative flow cytometry examples of HIV-GFP reporter expression in unstimulated live J-Lat 9.2 cells treated with 0.1% DMSO control (left), live cells stimulated to express HIV-GFP by 0.1 μg/mL of the control latency-reversing agent PMA (center), and suppression of PMA-induced virus expression in live cells by 10 μM hopeaphenol (right); (B) chemical structure of (–)-hopeaphenol; (C) effects of hopeaphenol on PMA-induced HIV production in J-Lat 9.2 cells. Blue dotted line denotes half-maximal inhibition. In panel C, results denote the mean ± SD from three independent experiments.

To identify new natural product-based inhibitors of HIV that act downstream of viral integration, we first used the J-Lat 9.2 cell line, which is derived from Jurkat T cells and contains a latent, noninfectious HIV provirus with a green fluorescent protein (GFP) reporter (16). In these cells, GFP expression indicates HIV transcription and viral antigen production. In the absence of stimulation, live-gated cells expressed little to no GFP, as monitored by flow cytometry (Fig. 1A). However, when stimulated for 24 h with 0.1 μg/mL of the PKC activator phorbol 12-myristate 13-acetate (PMA), we observed low-level but consistent GFP induction in an average of 10.7% ± 0.4% (mean ± standard deviation [SD]) of live-gated cells across all experiments (Fig. 1A).

Using this assay, we screened 512 compounds from the Davis open access library (14, 15) to identify those that inhibited PMA-induced GFP expression in live-gated J-Lat 9.2 cells at 10 μM following 24 h of incubation. The top hit from this screen was (–)-hopeaphenol (Fig. 1B), a stilbenoid previously isolated from the Anisoptera thurifera tree of Papua New Guinea (17). Hopeaphenol inhibited GFP expression in an average of 70.9% ± 3.0% live-gated cells compared to cells incubated with PMA alone (Fig. 1A). We also observed that hopeaphenol inhibited GFP in PMA-stimulated J-Lat 9.2 cells with dose dependence (Fig. 1C), with a calculated half-maximal effective concentration (EC_50_) of 1.8 μM. Hopeaphenol was therefore selected for further study.

### (–)-Hopeaphenol selectively inhibits HIV expression *in vitro*.

**FIG 2:**
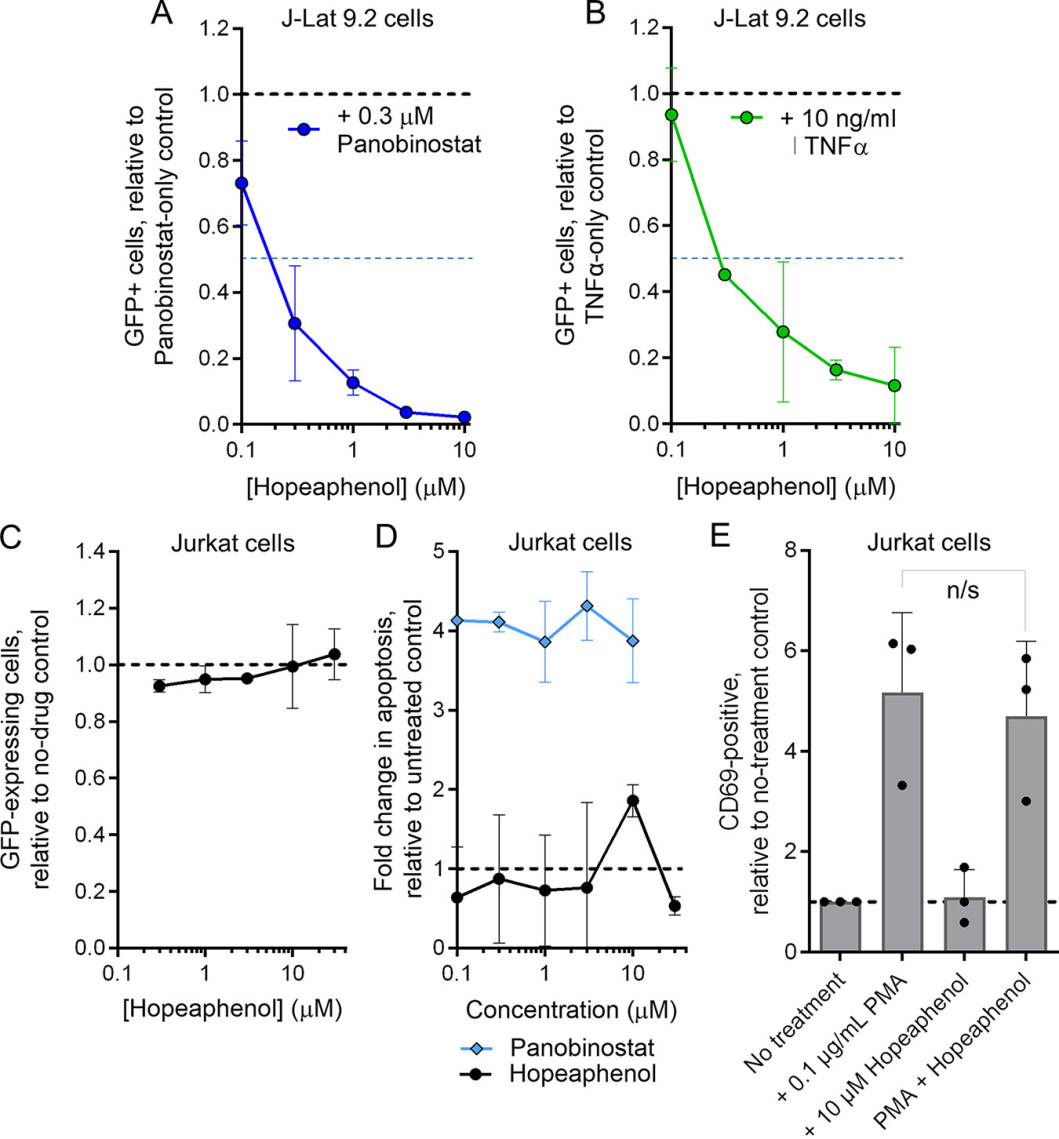
Effects of (–)-hopeaphenol on HIV production and cell viability *in vitro*. (A and B) Effects of hopeaphenol on HIV production in J-Lat 9.2 cells induced by panobinostat (blue [A]) or TNF-α (green [B]). The blue dotted line denotes half-maximal inhibition. (C) Effects of hopeaphenol on constitutive GFP production in transfected Jurkat cells; (D) effects of hopeaphenol and panobinostat on Jurkat cell apoptosis; (E) effects of hopeaphenol on PMA-induced CD69 expression in Jurkat cells. For each panel, data are presented normalized to cells without hopeaphenol treatment (black dotted lines). In all panels, results denote the mean ± SD from three independent experiments. n/s, not significant (*P* > 0.05) by one-sided *t* test.

We next determined if (–)-hopeaphenol could inhibit HIV expression induced by additional mechanisms of action. When J-Lat 9.2 cells were treated with 0.3 μM panobinostat, a histone deacetylase inhibitor, we observed GFP expression in 40.8% ± 15.9% of live-gated cells after 24 h, which was also inhibited by hopeaphenol with a calculated EC_50_ of 0.11 μM (Fig. 2A). Similarly, when cells were treated with 10 ng/mL of TNF-α, a proinflammatory cytokine and NF-κB stimulator, we observed GFP expression in 11.9% ± 5.9% of cells after 24 h, which was also subsequently inhibited by cotreatment with hopeaphenol (EC_50_ = 0.29 μM) (Fig. 2B), indicating that hopeaphenol could inhibit HIV expression regardless of proviral pathways targeted by these agents.

To confirm that hopeaphenol was not simply inhibiting GFP reporter fluorescence in this model, we transfected the parental Jurkat T-cell line with a plasmid that constitutively expresses GFP, which resulted in an average of 25.9% ± 2.3% GFP-positive cells observed by flow cytometry at 24 h following transfection. This GFP expression, however, was not inhibited by up to 30 μM hopeaphenol (average 1.0 ± 0.1-fold change in GFP expression versus untreated cells at 30 μM) (Fig. 2C), indicating that hopeaphenol does not directly inhibit detection of GFP expression and supporting that the loss of GFP observed above was due to lower HIV induction.

To confirm that loss of GFP expression was not due to cellular apoptosis, we next treated Jurkat T cells with hopeaphenol and assessed annexin V-positive cells by flow cytometry (Fig. 2D). While untreated cell cultures had a constitutive level of 10 to 15% annexin V-positive cells, no more than a 1.9 ± 0.2-fold increase in annexin V-positive cells was observed in the presence of 10 μM hopeaphenol (Fig. 2D), indicating limited cytotoxic effects with respect to induction of *in vitro* apoptosis. In contrast, treatment with 0.1 μM panobinostat, which is cytotoxic to J-Lat cells (18), induced an average 4.1 ± 0.1-fold increase in annexin V-positive Jurkat cells.

Finally, to determine whether hopeaphenol inhibited overall T-cell transcriptional activation that could be associated with HIV proviral expression, Jurkat T cells were treated with 0.1 μg/mL PMA, 10 μM hopeaphenol, or simultaneous 0.1 μg/mL PMA plus 10 μM hopeaphenol. After 24 h of incubation, cells were stained for the CD69 early T-cell activation marker, which showed an average 5.2 ± 1.6-fold increase in CD69^+^ cells by PMA alone over treatment with 0.1% dimethyl sulfoxide (DMSO) vehicle control, as expected (Fig. 2E). In contrast, no effect was observed on CD69 expression in cells treated with only hopeaphenol (i.e., 1.1 ± 0.6-fold change versus untreated cells) or induction levels by PMA in the presence of hopeaphenol (*P* = 0.06) (Fig. 2E), indicating that hopeaphenol did not immensely inhibit CD69 upregulation in T cells despite restricting provirus expression as shown above.

In summary, these results indicate that hopeaphenol selectively inhibits HIV proviral expression *in vitro* without inducing apoptosis or broadly inhibiting T-cell activation changes as indicated by CD69 upregulation following PMA stimulation.

### (–)-Hopeaphenol inhibits both viral transcription and a subset of T-cell activation pathways.

To identify changes in cellular RNA affected by (–)-hopeaphenol *in vitro*, we treated three independent preparations of J-Lat 10.6 cells, which resemble J-Lat 9.2 cells except for having provirus integration at a different genomic site, with 0.1% DMSO vehicle control, 0.1 μg/mL PMA, 10 μM hopeaphenol, or simultaneous 0.1 μg/mL PMA plus 10 μM hopeaphenol for 24 h. Following treatment, total RNA was extracted from cell cultures and assessed for global transcriptional changes by transcriptome sequencing (RNA-seq). For this study, J-Lat 10.6 cells were used due to their lower threshold for virus reactivation compared to other J-Lat cell lines (19); we anticipated this may result in larger HIV-dependent gene expression changes and thus clearer identification of hopeaphenol-dependent signaling pathways in T cells.

**FIG 3:**
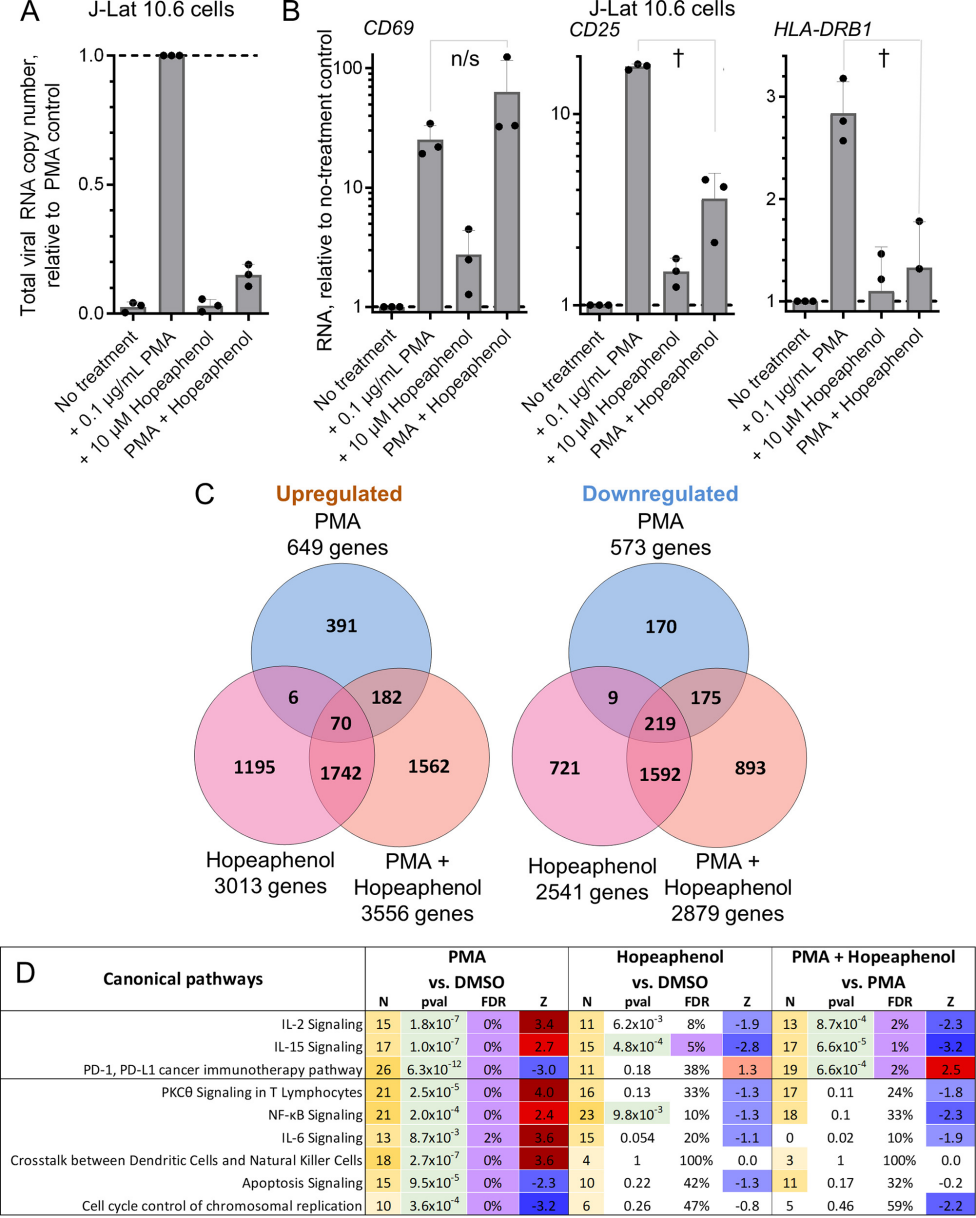
Effects of (–)-hopeaphenol on *in vitro* global gene expression as measured by RNA-seq. (A) Viral RNA copy number detected by RNA-seq from three independent cultures of J-Lat 10.6 cells treated with 0.1 μg/mL PMA, 10 μM hopeaphenol, or 0.1 μg/mL PMA plus 10 μM hopeaphenol; (B) effects of hopeaphenol on PMA-induced CD69 (left), CD25 (*IL2RA* [center]), and HLA-DRB1 (right) RNA expression across three J-Lat 10.6 cell cultures as detected by RNA-seq. n/s, not significant (*P* > 0.05), and †, *P* < 0.01, by one-sided *t* test. (C) Venn diagrams showing numbers of significantly upregulated or downregulated genes (false-discovery rate [FDR] of <5%) in J-Lat 10.6 cells treated under the conditions described for panel A compared to cells treated with 0.1% DMSO; (D) representative Ingenuity Pathway Analysis of genes identified in panel C that passed thresholds of an FDR of <5% and |Z-score| of >2. For each pathway, the data listed include the number of affected genes (N), *P* value (pval), FDR, and Z-scores (Z) for the predicted pathway state (where positive and negative values indicate activation or inhibition by treatment, respectively). The numbers of genes and Z-scores are highlighted by color scales.

High-quality sequence data were obtained from all samples, as >90% of reads aligned to the host genome in all cases. As expected, we observed that, compared to samples treated with PMA alone, samples treated with PMA plus hopeaphenol had reduced total cellular HIV RNA, with 85.1% ± 4.2% fewer viral RNA reads detected (Fig. 3A), confirming that hopeaphenol inhibits PMA-induced HIV transcription. Notably, the levels of CD69 RNA expression in these samples (Fig. 3B, left) approximated the effects on CD69 protein levels observed above in Jurkat cells (Fig. 2E), even despite the use of a different cell line containing HIV provirus here. For example, compared to untreated cells, J-Lat 10.6 cells treated with PMA or PMA plus hopeaphenol had 25.1 ± 8.1- and 63.2 ± 52.9-fold increases in CD69 RNA, respectively (*P* = 0.14) (Fig. 3B, left). In contrast, while PMA also induced a 17.7 ± 0.6-fold increase in the late T-cell activation marker CD25, cells treated with PMA and hopeaphenol had 3.6 ± 1.3-fold increased CD25 RNA, or a 78.9% decrease from PMA-only controls (*P* = 1.8 × 10^−3^) (Fig. 3B, center). Similarly, while PMA induced a 2.8 ± 0.3-fold increase in the human leukocyte antigen (class II) beta chain 1 RNA (HLA-DRB1) T-cell activation marker, cells treated with PMA and hopeaphenol had 1.3 ± 0.5-fold increased HLA-DRB1 RNA, or a 53.6% decrease from PMA-only controls (*P* = 1.8 × 10^−3^) (Fig. 3B, left). These results indicate that hopeaphenol inhibits a subset of makers that denote T-cell activation at the RNA level.

From these data sets, we also identified a total of 8,927 unique differentially expressed genes (based on a false-discovery rate [FDR] of <5%) that were affected by exposure to PMA and/or hopeaphenol compared to 0.1% DMSO-treated controls (Fig. 3C). The largest cluster of differentially expressed transcripts was detected due to treatment with hopeaphenol, where a total of 5,554 changes (3,013 upregulated + 2,541 downregulated) or 6,435 changes (3,556 upregulated + 2,879 downregulated) were seen in the absence or additional presence of PMA, respectively. Of these genes, 3,623 were shared among both groups (1,812 upregulated + 1,811 downregulated), representing 65.2% and 56.3% of total gene expression changes due to hopeaphenol and PMA plus hopeaphenol, respectively.

Genes affected by each treatment were then assessed for enrichment of canonical pathways using Ingenuity Pathway Analysis (19) (Fig. 3D; see Table S1 in the supplemental material). As expected for a PKC agonist, cells treated with PMA had distinct upregulation of genes involved in PKCθ signaling in T lymphocytes (Z-score = 4.0 [see Materials and Methods]) as well as NF-κB signaling (Z-score = 2.4). We also found that PMA induced (i) upregulation of interleukin-2 (IL-2), IL-6, and IL-15 signaling (Z-scores = 3.4, 3.6, and 2.7, respectively), (ii) downregulation of the PD-1/PD-L1 pathway (Z-score = −3.0), (iii) upregulation of the “cross talk between dendritic cell and natural killer cell” pathway (consisting of genes also underlying dendritic cell-mediated T-cell activation, such as CD28, CD69, HLA class I genes, TNF receptor genes, and others [Z-score = 3.7]) (20, 21), and (iv) downregulation of pathways involved in apoptosis signaling (Z-score = −2.3) and cell cycle control of chromosomal replication (Z-score = −3.2). These signaling pathways (Fig. 3D, left), among others (Table S1), are all consistent with PMA inducing a state of T-cell activation *in vitro*.

Compared to cells treated with 0.1% DMSO vehicle control, cells treated with hopeaphenol in the absence of PMA had significant downregulation only of genes involved in IL-15 signaling (Z-score = −2.8; FDR = 5%) (Fig. 3, center; Table S1). However, the overall directionality of gene expression changes tended to parallel those observed in cells treated with PMA plus hopeaphenol (Fig. 3D, center and right). These results indicated that while hopeaphenol induces gene expression changes (Fig. 3C), these do not result in statistically significant coordinated changes affecting broad-based cellular activation or cell death pathways.

In contrast, compared to PMA-treated samples, cells treated with PMA plus hopeaphenol showed distinct inhibition of a subset of PMA-induced pathways, including IL-15 signaling and IL-2 signaling (respective Z-scores = −3.2 and −2.3 relative to expression in PMA-treated cells) (Fig. 3D, right; Table S1), while the T-cell-inhibitory PD-1/PD-L1 pathway was upregulated (Z-score = 2.5). In contrast, changes in IL-6 signaling were not detected. Moreover, expression of the “cross talk between dendritic cells and natural killer cells” pathway also remained unaffected (Z-score = 0; FDR = 100%), consistent with persistent CD69 expression observed above. While gene expression involved in PKCθ and NF-κB signaling was reduced by hopeaphenol plus PMA relative to cells treated with only PMA, these changes did not reach statistical significance (FDR > 5%), suggesting that this pathway may not be fully inhibited by hopeaphenol. Finally, hopeaphenol did not significantly affect gene expression related to apoptosis signaling or cell cycle control of chromosomal regulation.

Taken together, the results indicate that hopeaphenol inhibits viral RNA transcription *in vitro* and can antagonize a subset of gene expression pathways that underlie PMA-induced T-cell activation. However, several T-cell activation and signaling pathways are not affected in the presence of hopeaphenol.

### Functional assessment of RNA expression changes induced by (–)-hopeaphenol.

**FIG 4:**
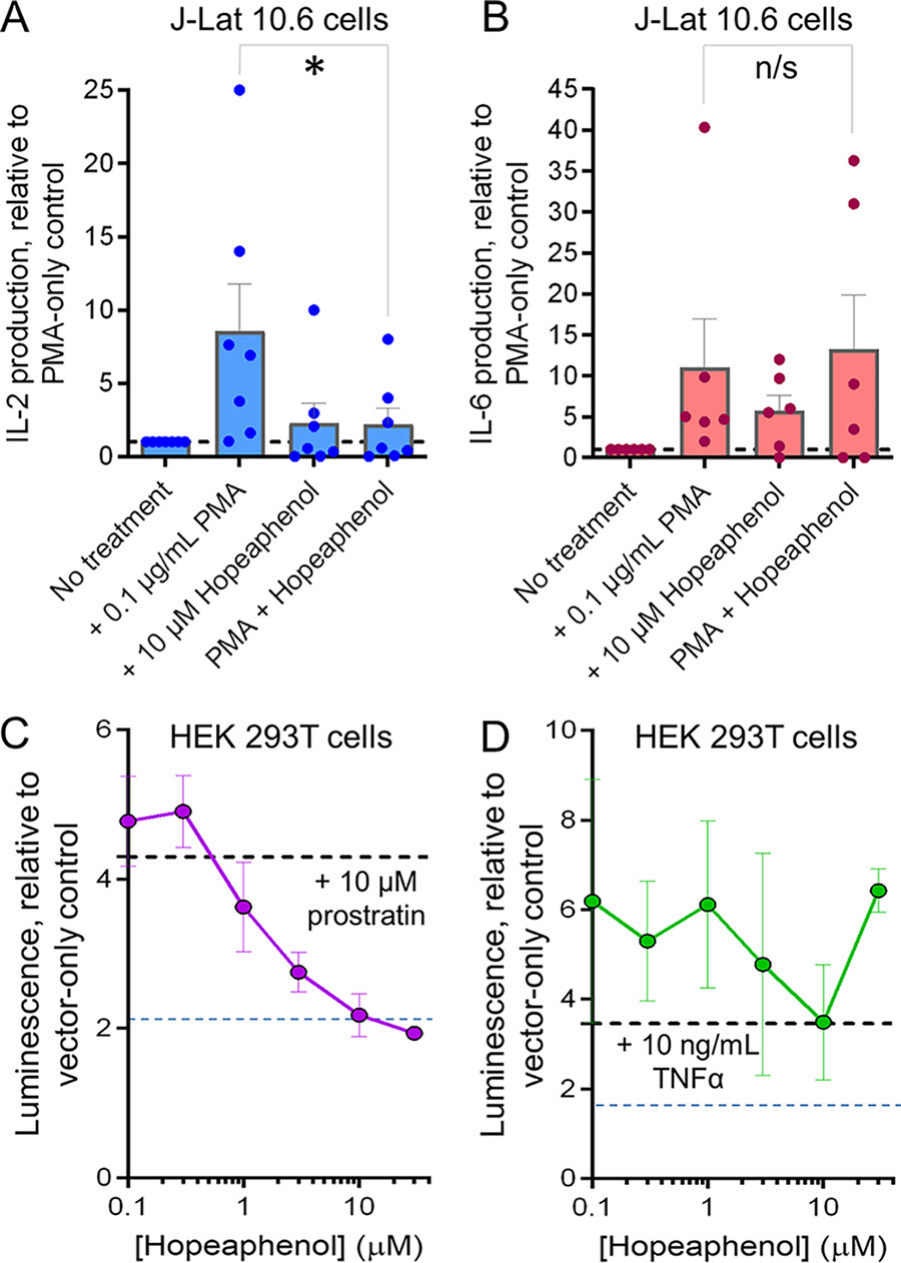
Effects of (–)-hopeaphenol on T-cell activation markers and cellular pathways. (A and B) Effects of 24 h of treatment with PMA, hopeaphenol, and PMA plus hopeaphenol on supernatant IL-2 (A) and IL-6 (B) levels in J-Lat 10.6 cells as measured by ELISA. Data are presented as fold change in cytokine levels relative to untreated cells. *, *P* < 0.05, and n/s, not significant (*P* > 0.05), by one-sided *t* test. (C and D) Effects of hopeaphenol on NF-κB-dependent reporter expression, where luminescence was measured in HEK-293T cells transfected with a DNA construct driving firefly luciferase under the control of five NF-κB response elements and stimulated with 10 μM prostratin (C) or 10 ng/mL TNF-α (D) in the presence of hopeaphenol. Data are presented as luminescence relative to transfected cells treated with 0.1% DMSO only and no prostratin or TNF-α stimulation (black dotted lines), while blue dotted lines denote half-maximal inhibition. In panels A and B, results denote the mean ± SEM from 7 and 6 independent experiments, respectively. In panels C and D, results denote the mean ± SD from 3 independent experiments.

We next asked whether changes in RNA expression observed following PMA and (–)-hopeaphenol treatment described above with regard to IL-2 and IL-6 signaling (Fig. 3D) corresponded to cytokine levels *in vitro*. To test this, we treated J-Lat 10.6 cells as described above and monitored supernatant levels of IL-2 and IL-6 by enzyme-linked immunosorbent assay (ELISA). Supernatants from cells treated with PMA had an average 8.6 ± 3.2-fold (mean ± standard error of the mean [SEM]) increase in IL-2 compared to supernatants from untreated cells (Fig. 4A). While hopeaphenol treatment on its own did not substantially affect supernatant IL-2 levels (2.3 ± 1.4-fold increase over untreated cells), it did reduce supernatant IL-2 levels in cells cotreated with PMA plus hopeaphenol by 74.3% compared to PMA-treated cells alone (i.e., 2.2 ± 1.1-fold over untreated cells; *P* = 0.013) (Fig. 4A), consistent with reduced IL-2 transcription and lower IL-2 signaling pathway signatures by RNA-seq. Conversely, while PMA increased supernatant IL-6 levels by 11.0 ± 6.0-fold (mean ± SEM) over untreated cells, cotreatment of PMA plus hopeaphenol did not inhibit IL-6 production, where supernatant IL-6 levels remained 13.3 ± 6.6-fold over untreated cells (+20.9%; *P* = 0.41) (Fig. 4B), again consistent with hopeaphenol not affecting IL-6 secretion or IL-6 signaling pathway RNA signatures in PMA-treated cells. However, we did observe that treatment with hopeaphenol on its own also increased IL-6 production by 5.8 ± 1.9-fold (mean ± SEM), in contrast to RNA-seq predictions (Fig. 4B). The source of this discrepancy is not immediately clear but could reflect IL-6 protein upregulation that occurs downstream of IL-6 RNA transcription.

As viral transcription is enhanced by PKC-mediated NF-κB signaling (7, 11, 12), and inhibitors of this process can block viral transcription (13, 22), we next investigated the degree to which NF-κB signaling is affected by the presence of hopeaphenol as predicted by RNA-seq. To test this, we transfected HEK-293T cells with a minimal promoter consisting of five NF-κB sites that drive expression of a firefly luciferase reporter. Reporter expression was then induced by treating cells with 10 μM prostratin, a PKC activator and PMA analogue (Fig. 4C). In transfected cells, prostratin induced luciferase luminescence by 4.3 ± 0.3-fold compared to that in unstimulated cells. Notably, when prostratin-stimulated cells were coincubated with hopeaphenol, a dose-dependent inhibition of luciferase luminescence was observed. However, this inhibition was incomplete, with only 55.0% inhibition of NF-κB-driven luciferase at 30 μM (EC_50_ = 10.0 μM) (Fig. 4C). This partial inhibitory effect by hopeaphenol stands in contrast to the much greater inhibition observed for PMA-induced HIV suppression by hopeaphenol in J-Lat 9.2 cells (EC_50_ = 1.8 μM) (Fig. 1C). Furthermore, when the same cells were stimulated with 10 ng/mL TNF-α (which, on its own induced a 3.5 ± 1.4-fold increase in luminescence), we observed no inhibition of NF-κB-driven luciferase expression with up to 30 μM hopeaphenol despite increased experimental variability (Fig. 4D), which also contrasted with our observations for 10-ng/mL TNF-α-induced HIV suppression by hopeaphenol in J-Lat 9.2 cells (EC_50_ = 0.29 μM) (Fig. 2B). These results indicate that while hopeaphenol is capable of abrogating PKC-induced NF-κB signaling and affecting HIV transcription containing two NF-κB sites, transcriptional sites with multiple NF-κB sites may result in partial inhibition by hopeaphenol and/or additional targets of hopeaphenol may be involved.

### (–)-Hopeaphenol inhibits Tat-driven LTR reporter expression and CDK9 enzymatic activity.

**FIG 5:**
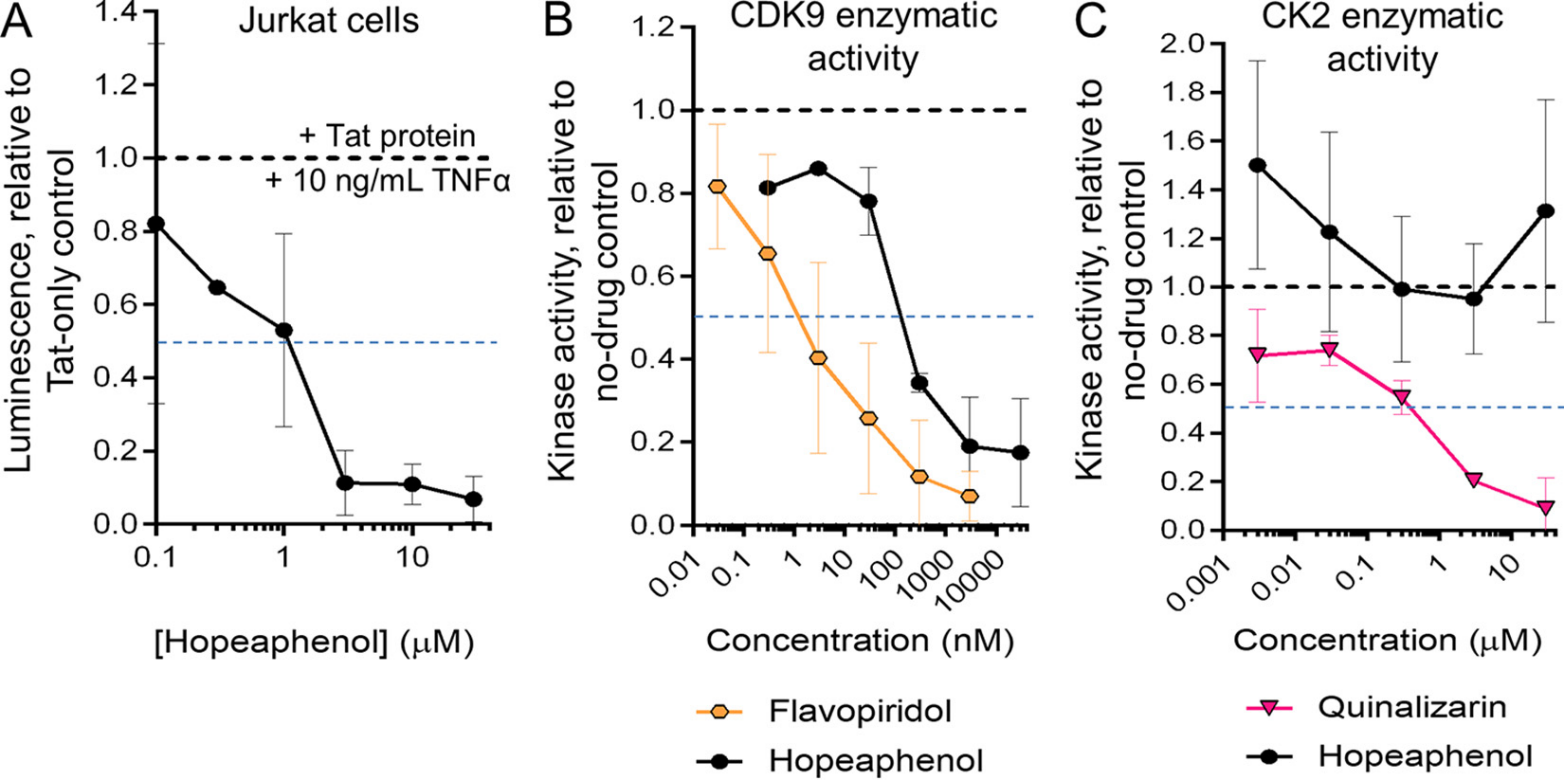
(–)-Hopeaphenol inhibits LTR-driven expression and CDK9 but not CK2 enzymatic activity. (A) Hopeaphenol inhibits Tat-driven LTR expression *in vitro*. Reporter luminescence was measured in Jurkat cells transfected with a cDNA construct encoding *Renilla* luciferase under the control of the HIV-1 LTR plus a constitutive Tat expression vector in the presence of 10 ng/mL TNF-α and hopeaphenol. Data are presented as luminescence relative to transfected cells treated with 0.1% DMSO and an empty control vector lacking Tat. (B and C) Enzymatic activities of CDK9 (B) and CK2 (C) in the presence of hopeaphenol or control inhibitors are shown. Data are presented relative to enzymatic activity in the presence of 0.1% DMSO. Data are presented relative to cells without hopeaphenol treatment (black dotted lines). Blue dotted lines denote half-maximal inhibition. In all panels, results denote the mean ± SD from three independent experiments.

Although data using a 5-NF-κB site-driven luciferase reporter suggest that TNF-α, and possibly some PKC activators, could drive expression despite the presence of (–)-hopeaphenol, contrasting earlier observations on the potential of hopeaphenol to inhibit HIV expression in the presence of both stimulations were also documented (Fig. 1C and Fig. 2C). To test whether hopeaphenol inhibits HIV transcription at the level of the LTR, we transfected Jurkat cells with a plasmid containing an HIV LTR promoter driving *Renilla* luciferase in the additional presence of 10 ng/mL TNF-α. In these cells, reporter expression was then induced by the cotransfection of a plasmid constitutively expressing Tat (Fig. 5A) (13). In cells transfected with Tat and treated with TNF-α, we observed a 45.1 ± 17.4-fold increase in luciferase luminescence relative to cells transfected with a control vector lacking Tat and without TNF-α. However, when Tat-transfected plus TNF-α-treated cells were additionally incubated with hopeaphenol, this luminescence was clearly inhibited, with an EC_50_ of 0.98 μM (Fig. 5A). Together with effects on host gene expression as noted above, these results indicate that hopeaphenol can inhibit LTR-mediated transcriptional expression even in the presence of TNF-α and constitutively expressed viral Tat.

As hopeaphenol’s structure consists essentially of two planar moieties (Fig. 1B), we next hypothesized that, like other planar compounds, it might also interfere directly with the active sites of kinases such as CDK9 to block virus transcription (23). To test this, we used an enzymatic assay and a luminescence reporter to quantify the levels of ATP converted to ADP by cell-free, purified CDK9-cyclin T1 complexes. Using this approach, we observed that the control CDK9 inhibitor flavopiridol blocked enzymatic activity, with a half-maximal inhibitory concentration (IC_50_) of 0.0029 μM (Fig. 5B), consistent with previous observations (23). We further observed that hopeaphenol used singly also inhibited CDK9 at an IC_50_ of 0.22 μM. In contrast, hopeaphenol did not inhibit the enzymatic activity of casein kinase 2 (CK2) (Fig. 5C), even despite increased experimental variability, unlike the control CK2 inhibitor quinalizarin (IC_50_ = 0.68 μM) (24), indicating that hopeaphenol selectively inhibits CDK9 over CK2.

Taken together, the results show that inhibition of LTR-mediated transcription downstream of TNF-α signaling and Tat-driven transcription is associated with the ability of hopeaphenol to directly inhibit CDK9 enzymatic activity.

### (–)-Hopeaphenol inhibits HIV replication in PBMCs and dampens viral reactivation in CD4^+^ T cells from PLWH.

**FIG 6:**
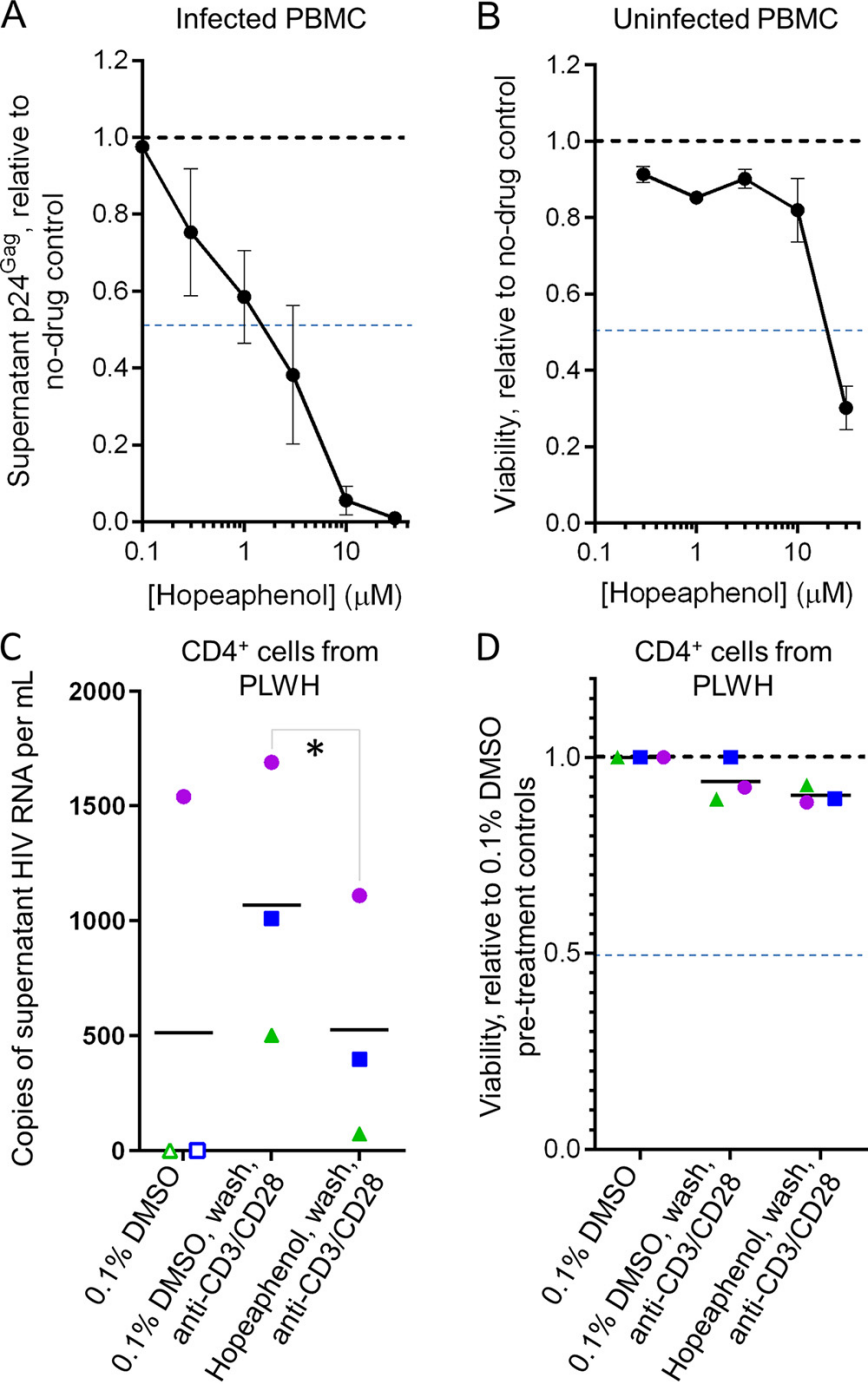
(–)-Hopeaphenol inhibits multicycle HIV replication in PBMCs and dampens viral reactivation without cytotoxicity in CD4^+^ T cells from PLWH. (A) Effects of hopeaphenol on HIV-1_NL4.3_ replication in PBMCs from four independent donors, as measured by p24^Gag^ levels at 6 days postinfection, as measured by ELISA; (B) effects of hopeaphenol on viability of uninfected PBMCs after 6 days in culture. For panels A and B, data are presented relative to cells without hopeaphenol treatment (black dotted lines), while blue dotted lines denote half-maximal inhibition. Results denote the mean ± SEM from PBMCs from four donors. (C) Effects of hopeaphenol pretreatment for 24 h on anti-CD3/CD28 Dynabead-induced viral reactivation in CD4^+^ T cells from three PLWH, as measured by supernatant viral RNA production. (D) Effects of treatments on CD4^+^ T-cell viability from the same three PLWH. For panels C and D, shapes and colors denote individual donors, and open symbols indicate no viral RNA detected. *, *P* < 0.05 by one-sided *t* test.

To confirm that (–)-hopeaphenol can act on *de novo* HIV infection and replication, activated PBMCs from four donors were infected *in vitro* with HIV-1_NL4.3_ for 6 h, washed, incubated with hopeaphenol for a total of 6 days, and monitored for production of supernatant p24^Gag^ viral antigen by ELISA as described previously (13). While donor-to-donor variability was consistent with previous observations by us (13, 23) and others (25, 26), we observed that hopeaphenol inhibited p24^Gag^ production, with an EC_50_ of 1.3 μM (Fig. 6A). Similarly, no major changes in uninfected PBMC viability from the same four donors were noted in the presence of hopeaphenol, as measured using Viacount dye (13), except when treating with the maximal dose of 30 μM, where a 69.8% ± 11.4% reduction in viability was observed (Fig. 6B). These observations corresponded to a calculated half-maximal cytotoxic concentration (CC_50_) of 19.3 μM or a selectivity index (CC_50_/EC_50_) of 14.8, indicating antiviral activity at low micromolar concentrations without concomitant cytotoxicity.

To investigate whether (–)-hopeaphenol may affect viral reactivation *ex vivo*, we next monitored effects of hopeaphenol on HIV RNA expression in cultures of CD4^+^ T cells isolated from three PLWH stably suppressed on cART (Fig. 6C and D). In these studies, CD4^+^ T cells were cultured in the presence of 10 μM hopeaphenol or 0.1% DMSO vehicle control for 24 h, at which point cells were extensively washed before inducting global T-cell activation with anti-CD3/CD28 Dynabeads. HIV reactivation was then monitored by supernatant viral RNA production using quantitative reverse transcription-PCR (qRT-PCR) as described previously (23). Following this time course, we detected spontaneous viral RNA production in vehicle-treated cells from only one of three donor samples, where 1,540 viral RNA copies per mL of cell culture were observed (Fig. 6C, filled circles). For the other two donor samples, viral RNA was below the limit of detection of 20 copies/mL (Fig. 6C, open symbols). In vehicle-treated cells subsequently stimulated with anti-CD3/CD28, we observed an average of 1,068 ± 543 viral RNA copies per mL across all samples (Fig. 6C). In contrast, when the same cells were pretreated with hopeaphenol, we observed a reduction in subsequent anti-CD3/CD28-induced viral RNA that corresponded to an average of 527 ± 531 viral RNA copies per mL, or a 50.7% reduction (*P* = 0.011) (Fig. 6C). No substantial effects on cell viability were observed following treatment regimens, as measured by direct cell counting with trypan blue, with no more than a 9.7% ± 2.3% reduction in cell number in cells pretreated with hopeaphenol and stimulated relative to unstimulated, vehicle-treated cells: the average cell densities were (2.4 ± 0.5) × 10^6^, (2.3 ± 0.3) × 10^6^, and (2.2 ± 0.5) × 10^6^ cells/mL in vehicle-pretreated, vehicle-pretreated and stimulated, and hopeaphenol-pretreated and stimulated cells, respectively (Fig. 6D). Taken together, these results show that hopeaphenol pretreatment can dampen subsequent viral reactivation in CD4^+^ T cells from PLWH.

## DISCUSSION

Despite effective viral suppression that prevents progression to AIDS, PLWH on cART continue to harbor infected cells capable of ongoing viral RNA expression and antigen production. While the recent advent of long-acting antiretrovirals creates promising opportunities to improve cART adherence and reduces the probability of cART-related long-term toxicities (27), these therapies likely remain insufficient to suppress ongoing HIV transcription and production of viral antigens potentially linked to secondary consequences of chronic inflammation and age-related comorbidities. Toward the long-term goal of developing novel chemical leads to address constitutive cell-associated viral RNA expression and viral antigens in spite of cART, we identify (–)-hopeaphenol as an inhibitor of viral transcription and viral replication in cell lines and primary cells as well as a dampener of viral reactivation from CD4^+^ T cells from PLWH, as identified from a starting pool of 512 pure compounds derived from natural products.

Hopeaphenol and structurally related plant stilbenoids are reported to possess numerous *in vitro* properties, including antiproliferative (28–31), antibacterial (17, 32), and anti-inflammatory (33) activities. Hopeaphenol has also been shown to inhibit replication of influenza A and herpes simplex viruses 1 and 2 at low micromolar concentrations without cytotoxicity *in vitro*, although the mechanisms of action are not yet fully defined (34). More recently, we reported that hopeaphenol inhibits replication of multiple severe acute respiratory syndrome coronavirus 2 (SARS-CoV-2) variants at low micromolar concentrations *in vitro* through antagonizing the viral spike glycoprotein interaction with its cellular ACE2 receptor (35). These previous results, combined with those described here, support the hypothesis that hopeaphenol is likely a broad-spectrum natural antiviral compound. Structurally, hopeaphenol is a tetramer of resveratrol molecules, and interestingly both resveratrol and resveratrol analogs are reported to promote HIV latency reversal and viral expression through a variety of mechanisms, including stimulating histone acetylation, heat shock factor 1 expression, CDK9 phosphorylation, p-TEFb activation, and EGR1 expression (36–38). Thus, further structure-activity relation studies with both stilbenoid and resveratrol analogues may identify chemical moieties that underlie both HIV latency reversal and HIV silencing in T cells.

**FIG 7:**
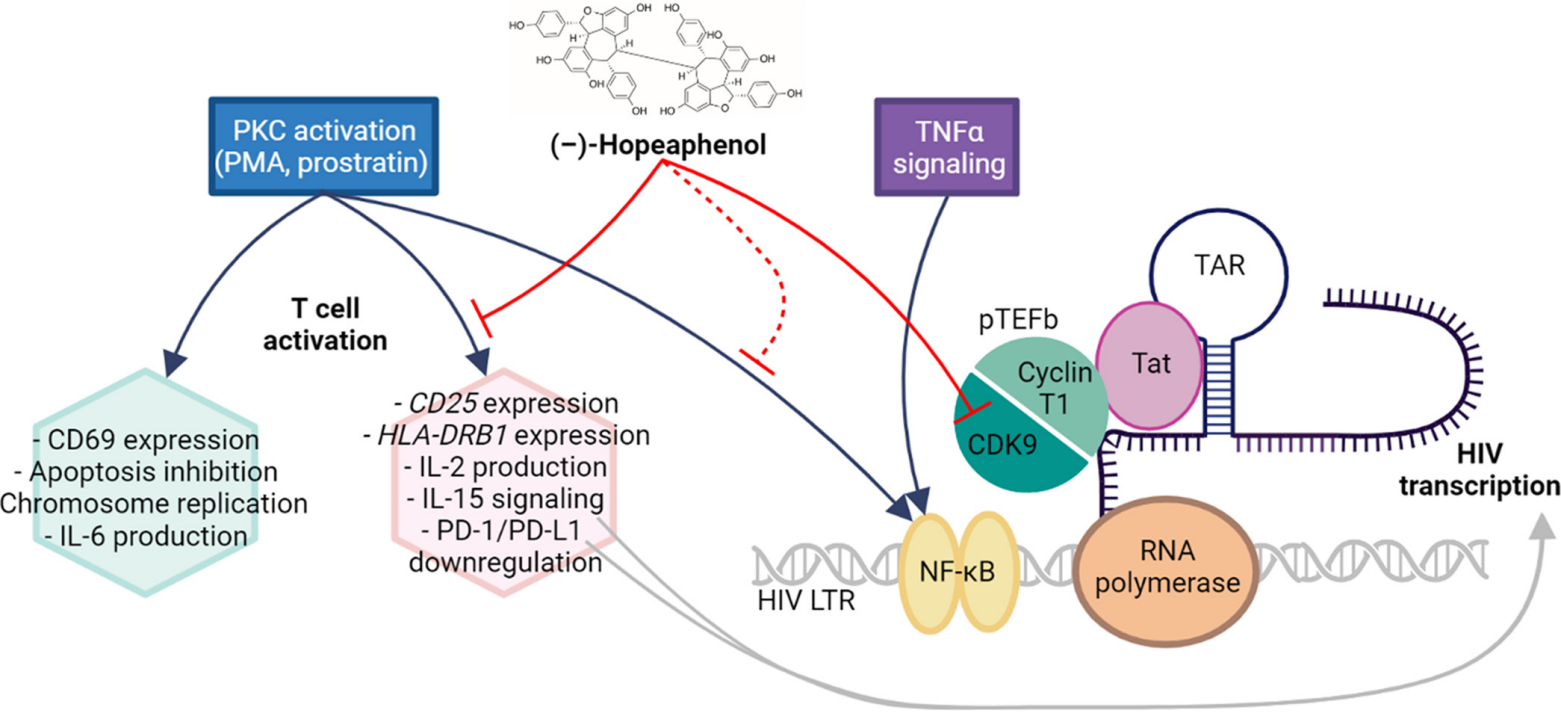
Summary of T-cell and HIV activation pathways targeted by (–)-hopeaphenol. The figure was created with BioRender.com.

An important consideration of hopeaphenol is that, as a polyphenolic compound, it falls within the structural classes with a PAINS (pan assay interference compounds) designation (39) and thus potential nonspecific activities against multiple biological targets. However, our results instead support that hopeaphenol selectively acts on a subset of biological processes to promote antiviral activity without obviously affecting cell homeostasis or cell viability. For example, we show here that hopeaphenol does not inhibit constitutive GFP expression in cell lines, in contrast to inhibiting GFP-tagged viral reactivation in cell lines. We also show that only a subset of T-cell activation targets induced by PMA are antagonized by hopeaphenol (see below) (Fig. 7), indicating that hopeaphenol acts on distinct biological pathways. Hopeaphenol was also able to block enzymatic activity of CDK9 but not that of CK2. Finally, we show that hopeaphenol blocks HIV replication in PBMCs and dampens viral reactivation in latently infected CD4^+^ T cells without concomitant cytotoxicity. Taken together, these results are more consistent with a model in which hopeaphenol has a broad but selective range of cellular and viral targets that collectively act to antagonize viral reactivation and HIV transcription (Fig. 7). Notably, *in vivo* studies also report that hopeaphenol is tolerated in mice at up to 200 mg/kg (40, 41), indicating host tolerance to this compound. As a result, future efficacy studies of hopeaphenol-induced viral suppression in humanized mouse models of HIV infection may be possible. These studies might also be able to test directly whether hopeaphenol, as a supplement to cART, can also dampen markers of chronic immune activation and/or enhance immune reconstitution in infected, humanized mice compared to infected animals treated with cART alone.

The ability of hopeaphenol to inhibit HIV production was demonstrated here using both cell lines and primary cells, where comparable results were observed, including EC_50_s in the low- to submicromolar range. Hopeaphenol also inhibited virus production induced by several proviral agents *in vitro*, including PMA, panobinostat, and TNF-α. When J-Lat cells were treated with PMA plus hopeaphenol and monitored for global RNA expression, we observed an 85.1% ± 4.2% reduction in viral RNA reads relative to cells treated only with PMA, thus directly demonstrating that hopeaphenol inhibits viral transcription. In contrast, while hopeaphenol did inhibit PMA-induced CD25 and HLA-DRB1 RNA expression, as assessed by RNA-seq, it did not immensely affect PMA-induced CD69 RNA expression in J-Lat 10.6 cells or CD69 protein expression in Jurkat cells (Fig. 7), indicating that hopeaphenol is unlikely to act simply as a broad inhibitor of T-cell activation. These observations were further supported by the ability of hopeaphenol to inhibit PMA-induced IL-2 but not IL-6 production. RNA-seq studies also indicated that a subset of T-cell activation pathways affected by PMA were also antagonized by hopeaphenol: for example, IL-15 signaling, where resulting downregulation by hopeaphenol is consistent with blocking HIV progression (42), and PD-1/PD-L1 signaling, where upregulation by hopeaphenol is consistent with reinforcing HIV latency (43) (Fig. 7, gray arrows). Although more mechanistic studies are needed, these gene expression changes induced by PMA and suppressed by hopeaphenol are consistent with suppressing HIV expression (Fig. 7). Future studies will also need to support how these gene expression changes may impact T-cell functionality, even if viability was not obviously affected at concentrations able to inhibit HIV RNA expression.

In contrast, other T-cell activation pathways induced by PMA remained preserved in the presence of hopeaphenol as measured by RNA-seq (Fig. 7). For example, as indicated by both RNA-seq and *in vitro* observations, we observed that IL-6 production induced by PMA was not affected by hopeaphenol. We also saw that NF-κB signaling stimulated by TNF-α and, to some extent, PKC activation, retained at least some functionality in the presence of hopeaphenol. These results are consistent with a recent study showing that (+)-hopeaphenol can partially inhibit poly(I-C)-induced phosphorylation of the p65 component NF-κB but not that of the IRF3 (interferon regulatory protein 3) transcription factor (44). However, while hopeaphenol was unable to completely dampen cellular NF-κB-mediated transcription, as modeled by a 5-NF-κB site construct, our data directly confirm that LTR-mediated reporter expression driven by constitutive Tat and TNF-α was potently inhibited by hopeaphenol, with EC_50_s similar to those observed in J-Lat T cells. In addition, in enzymatic assays, hopeaphenol inhibited CDK9 but not CK2 enzymatic activity. These results are consistent with the essential role of CDK9, as part of the P-TEFb complex, in RNA polymerase II phosphorylation at the LTR for productive HIV transcription (8–10). They are also consistent with prior work demonstrating that CDK9 inhibition can antagonize postintegration HIV expression without a major effect on cell homeostasis (23, 45). Taken together, these results support the idea that the effects of hopeaphenol cannot be overcome by the HIV-1 clade B isolates tested here, at least *in vitro*. It remains to be determined, however, if an equal inhibition would be present in HIV-1 clade C isolates, where additional NF-κb sites are present within the viral LTR (46–48).

Interestingly, hopeaphenol pretreatment, followed by its discontinuation, was able to dampen subsequent viral reactivation in CD4^+^ cells from cART-suppressed PLWH. While numerous compounds have been reported to silence HIV expression, only a handful are reported to maintain this silencing following discontinuation and/or following subsequent proviral stimuli. Among the best-characterized inhibitors of this so-called “block-and-lock” class of agents is didehydro-cortistatin A (dCA), a natural product derivative that binds the Tat basic domain and interferes with Tat’s ability to recruit the p-TEFb complex to the viral TAR element for productive HIV transcription (49). Our observations that hopeaphenol targets both CDK9-dependent virus expression as well as a subset of proviral T-cell activation targets (Fig. 7) raise the possibility that hopeaphenol might be able to act in combination with dCA or related agents to accelerate viral silencing and/or enhance the duration of silencing following discontinuation, although additional studies are clearly needed to explore these possibilities.

There are currently no licensed antiretrovirals that directly inhibit viral transcription and viral antigen production or maintain viral silencing in infected cells. By acting to inhibit Tat-driven HIV transcription, hopeaphenol could be a useful lead toward identifying therapeutic candidates or combination therapies that can be developed to durably and/or permanently suppress ongoing cell-associated viral RNA and antigen production in the presence of cART in PLWH.

## MATERIALS AND METHODS

### Cells, compounds, and reagents.

Jurkat T cells (clone E6-1) were obtained from the American Tissue Culture Collection. HEK-293T cells (Lenti-X 293T) were obtained from TaKaRa Bio (Mountain View, CA, USA). The following reagents were obtained from the NIH AIDS Reagent Program: J-Lat T-cell clones 9.2 and 10.6 (from Eric Verdin) (16) and pNL4-3 (from M. Martin) (50). PBMCs were obtained from four uninfected donors following written informed consent. Study protocols were approved by the Institutional Review Boards of Simon Fraser University and the University of British Columbia—Providence Health Care Research Institute. CD4^+^ T cells were separately obtained from three PLWH on stably suppressive cART (plasma viral load of <50 copies/mL) for at least 3 years following written informed consent. These participants were recruited in accordance with human subject research guidelines of the U.S. Department of Health and Human Services under the supervision of the Wistar and Philadelphia FIGHT institutional review boards.

Primary cells and T-cell lines were cultured in R10+ medium (RPMI 1640 with HEPES and l-glutamine, 10% fetal bovine serum [FBS], 100 U of penicillin/mL, and 100 μg of streptomycin/mL [Sigma-Aldrich, St. Louis, MO, USA, and Oakville, ON, Canada]). T cells from PLWH were cultured in R20+ medium, which is identical to R10+ except for 20% FBS. HEK-293T cells were cultured in Dulbecco’s modified Eagle’s medium with 4.5 g/L glucose and l-glutamine (Lonza, Basel, Switzerland) with FBS and antibiotics as described above.

The Davis open access natural product-based library consists of 512 distinct compounds, the majority (53%) of which are natural products obtained primarily from Australian fungal, plant, and marine invertebrate sources (14, 15), as well as semisynthetic natural product analogues (28%) and known commercial drugs or synthetic compounds inspired by natural products (19%). All compounds evaluated in this study were analyzed for purity prior to testing and shown to be >95% pure. Compounds were initially provided by Compounds Australia at Griffith University in 5 mM stock solutions dissolved in dimethyl sulfoxide (DMSO) (Millipore, Burlington, MA, USA); as such, DMSO was used as the vehicle control in this study. (–)-Hopeaphenol was resupplied as dry powder for confirmation studies and further biological evaluation.

The isolation, structural confirmation, and purity of the (–)-hopeaphenol used in this study were reported previously (17). PMA, prostratin, TNF-α, panobinostat, quinalizarin, and human IL-2 were obtained from Sigma-Aldrich with reported ≥95% purity. ADP-Glo kinase assays and purified CK2α were obtained from Promega (Madison, WI, USA), and purified CDK9-cyclin T1 complex was obtained from Sigma-Aldrich. pSelect-GFP expression plasmid was obtained from InvivoGen (San Diego, CA, USA). PathDetect pNF-κB-Luc Cis-Reporter plasmid (pNF-κB-Luc) was obtained from Agilent (Santa Clara, CA, USA). pCMV-tat (an HIV-1 Tat expression vector), pCMV-Δtat (its corresponding control lacking Tat), and pLTR-RL (an HIV-1 LTR-driven *Renilla* luciferase reporter) were previously described (51, 52) and were provided as a gift from Brendan Bell.

### Provirus expression assays.

J-Lat 9.2 or 10.6 cells were seeded in 96-well plates at 2 × 10^5^ cells/well and treated in duplicate in the presence of test agents at defined concentrations or 0.1% DMSO vehicle control for 24 h. Live-gated cells were then examined for GFP expression by flow cytometry.

### Cellular GFP quenching assay.

A total of 5 × 10^6^ Jurkat cells were resuspended in 200 μL of Opti-MEM (Thermo Fisher Scientific, Waltham, MA, USA) containing 5 μg of pSelect-GFP. Cells were transfected in 0.4-cm cuvettes using a GenePulser MXCell electroporation system (Bio-Rad, Hercules, CA, USA) with a single 250-V square wave pulse for 25 ms. Cells were rested for 10 min at room temperature prior to seeding into 96-well plates at 10^5^ cells/well and then were treated with test agents or 0.1% DMSO control. After 24 h, GFP expression was detected by flow cytometry.

### Viability and cell activation detection assays.

A total of 2 × 10^5^ Jurkat cells were seeded into 96-well plates and treated in duplicate with test agents for 24 h. Following incubation, cells were stained with annexin V-allophycocyanin (APC) according to the manufacturer’s instructions (BioLegend, San Diego, CA, USA) and monitored by flow cytometry. To measure cell activation, Jurkat cells were stained with CD69-phytoerythrin (BD Biosciences, Mississauga, ON, Canada) antibody using the Cytofix/Cytoperm fixation/permeabilization kit (BD Biosciences) according to the manufacturer’s instructions. Flow cytometry was then performed as described above.

### RNA-seq and data analysis.

Two million J-Lat 10.6 cells were incubated in 2 mL of R10+ medium and treated in triplicate with test agents for 24 h. RNA-seq and data analysis were then performed as described previously (19). RNA was extracted from cells using the AllPrep DNA/RNA/miRNA universal kit (Qiagen; Germantown, MD, USA) with on-column DNase treatment (Qiagen RNase-free DNase set). One hundred nanograms of DNase-treated total RNA was used for library preparation with the Quant-Seq 3′ mRNA-Seq library preparation kit (Lexogen, Vienna, Austria). Library quantity was determined by quantitative PCR (qPCR) (KAPA Biosystems, Inc., Wilmington, MA, USA), and library size was determined using the Agilent TapeStation and DNA high-sensitivity D5000 ScreenTape (Agilent). Libraries were pooled in equimolar amounts and denatured, and single-read, 75-bp sequencing was performed using a NextSeq 500 (Illumina, San Diego, CA).

RNA-seq data were aligned against the human genome (v.hg19) using STAR (53). Raw read counts were estimated using RSEM v.1.2.12 software (54) with Ensemble transcriptome information v.GRCh37.13. Raw counts were normalized and tested by DESeq2 (55) to determine significance of differential expression, defined as genes that passed the false-discovery rate (FDR) of <5%. Gene set enrichment analysis was performed with Ingenuity Pathway Analysis software (Qiagen) using the “canonical pathways” category. Nominal *P* values were adjusted for multiple testing using the Benjamini-Hochberg procedure (56). Pathways enriched at an FDR of <5% and with a predicted activation |Z-score| of >2 in at least one treatment were determined.

### Cytokine ELISAs.

Two million J-Lat 10.6 cells were incubated in 2 mL R10+ plus test agents for 24 h in triplicate. Supernatants were then collected and assessed for IL-2 and IL-6 using human IL-2 and IL-6 ELISA kits (Abcam, Boston, MA, USA) according to the manufacturer’s instructions.

### NF-κB-based reporter assays.

HEK-293T cells were seeded at 70% confluence and allowed to attach overnight. Cells were then transfected with 5.5 μg of pNF-κB-Luc using Lipofectamine LTX. After 48 h, cells were seeded into 96-well plates at 10^4^ cells/well and treated with 0.1% DMSO or test agents. Following 24 h of incubation, firefly luciferase was detected using the Dual-Glo luciferase assay system (Promega) on an Infinity M200 multimode plate reader (Tecan Life Sciences; Männedorf, Switzerland). Data were normalized to luminescence from 0.1% DMSO-treated cells.

### HIV-1 LTR-based reporter assays.

A total of 5 × 10^6^ Jurkat cells were resuspended in 200 μL of Opti-MEM containing 8.4 μg of pLTR-RL plus 1.6 μg of either pCMV-Tat or pCMV-Δtat. Cells were transfected using the GenePulser MXCell electroporation system as described above. Following recovery for 10 min, 96-well plates were seeded with 5 × 10^4^ cells/well and incubated with test agents plus 10 ng/mL TNF-α. After 24 h, *Renilla* luciferase activity was monitored using the Dual-Glo luciferase assay system. Data were normalized to luminescence of DMSO-treated cells transfected with pLTR plus pCMV-Tat. Cells transfected with pLTR plus pCMV-Δtat served as a negative control to confirm Tat-driven luciferase from the LTR plasmids.

### Kinase activity assays.

Kinase reactions were performed in white 384-well plates, with 10-μL final volumes per well, following the manufacturer’s instructions (ADP-Glo kinase assay kit; Promega). Reagents and flavonoids were diluted in the supplied kinase reaction buffer. For CDK9-cyclin T1 assays, 30 ng of CDK9-cyclin T1 was incubated with 10 μM ATP, 0.2 μg/mL PDKTide peptide substrate, and test agents. For CK2 assays, 10 ng of CK2α was incubated with 10 μM ATP, 0.1 μg/mL casein peptide substrate, and test agents. No-enzyme and no-inhibitor control conditions were also included. After 40 min, kinase reactions were stopped by addition of 10 μL/well ADP-Glo reagent, and mixtures were incubated at room temperature for an additional 40 min. Twenty microliters per well of kinase detection reagent was then added, and luminescence intensity was measured on an Infinity M200 multimode plate reader after 30 min. The resulting data were normalized to range between 0 and 1, with no-enzyme and no-inhibitor conditions representing 0 and 1, respectively.

### Virus production.

HEK-293T cells were transfected with 15 μg of pNL4-3 via Lipofectamine LTX (Invitrogen; Burlington, ON, Canada) according to the manufacturer’s instructions. Cell culture supernatants containing HIV-1_NL4.3_ virions were harvested after 48 h and stored at −80°C. Viral titers were determined as described previously (57).

### HIV replication in PBMCs.

PBMCs from 4 donors without HIV were activated for 3 days with 5 μg/mL phytohemagglutinin in R10+ medium, followed by infection with HIV-1_NL4.3_ for 6 h (multiplicity of infection [MOI] = 0.003). PBMCs were then pelleted and resuspended in R10+ medium supplemented with 100 U/mL IL-2, seeded into 96-well U-bottom plates at 1.5 × 10^5^ cells/well, and treated with test agents or 0.1% DMSO. On day 3 postinfection, media, test agents, and IL-2 were replenished. Supernatant p24^Gag^ was quantified on day 6 postinfection by ELISA (Xpress Bio, Frederick, MD, USA).

Viability of PBMCs was assessed using the procedure described above, with uninfected PBMCs from the same donors. Following 6 days posttreatment, culture viability was assessed using Guava Viacount dye (Luminex; Toronto, ON, Canada) and flow cytometry as described previously (13).

### Measures of viral RNA production in primary CD4^+^ T-cell cultures.

CD4^+^ T cells from PLWH were isolated from PBMCs by negative selection using the EasySep human CD4^+^ T-cell enrichment kit (STEM-CELL Technologies, Vancouver, BC, Canada) and cultured as described previously (19). A total of 10^6^ cells were then treated with 0.1% DMSO or test agents for 24 h in 1-mL cultures in R20+ plus 100 U/mL IL-2. Cells were then washed extensively and resuspended in fresh R20+ plus IL-2 and 0.1% DMSO in the presence of anti-CD3/CD28 Dynabeads (Invitrogen) in a 1:1 ratio for 24 h. Following incubation, live cells were counted visually by trypan blue staining. Viral RNA was extracted from culture supernatants using a QIAmp viral RNA minikit (Qiagen) and subjected to quantitative PCR, as described previously, using a C1000 thermal cycler and CFX96 real-time system (Bio-Rad) (58). The limit of detection for supernatant viral RNA was 20 copies/mL as determined through endpoint detection from serial dilution of the AcroMetrix HIV-1 panel (Thermo Fisher).

### Data and statistical analyses.

Flow cytometry data were analyzed using FlowJo v.10.5.3 (FlowJo LLC. Ashland, OR, USA). Background GFP in unstimulated J-Lat 9.2 cultures and mock-transfected Jurkat cells was set to 0.05%. Half-maximal effective and cytotoxic concentrations (EC_50_s and CC_50_s, respectively) were calculated using the linear regression function in GraphPad Prism v.8.2.1 (GraphPad, San Diego, CA, USA). For assays assessing inhibition of provirus expression, compound EC_50_s were calculated after normalizing results from treated cells to those of latency reversing agent-only controls. All data are presented as mean ± SD (or SEM where stated) from at least 3 independent experiments or from at least 3 primary cell donors. Statistical significance was determined using the paired *t* test, where a one-sided *P* value of <0.05 was considered significant.
