# Supplementary material for: The Natural Stilbenoid (–)-Hopeaphenol Inhibits HIV Transcription by Targeting Both PKC and NF-κB Signaling and Cyclin-Dependent Kinase 9
Source: Antimicrob Agents Chemother. 2023 Mar 15;67(4):e01600-22. doi: 10.1128/aac.01600-22 (PMC10112218; doi:10.1128/aac.01600-22)
Supplement: Supplemental file 1 — Supplemental material. Download aac.01600-22-s0001.docx, DOCX file, 0.03 MB [file aac.01600-22-s0001.docx]

**Table S1.** Total Ingenuity Pathway Analysis of genes identified in **Fig. 3C** that passed FDR < 5% and |Z-score| > 2 thresholds in comparisons of PMA-treated vs. untreated cells, (–)-hopeaphenol-treated vs. untreated cells, and PMA plus (–)-hopeaphenol vs. PMA-treated cells. For each pathway, data listed include Z-scores (Z) for predicted pathway state (where positive and negative values indicate activation or inhibition by treatment, respectively), number of affected genes (N), p value (pval) and FDR.

| **Supplemental Table 1** |  |  |  |  |  |
| --- | --- | --- | --- | --- | --- |
| **PMA vs. No treatment** | | | | | |
| **Signaling pathway** | **p** | **FDR** | **State** | **Z** | **N genes** |
| PD-1, PD-L1 cancer immunotherapy pathway | 6.30957E-12 | 0% | Inhibited | -3 | 26 |
| IL-9 Signaling | 2.0893E-09 | 0% | Activated | 3.051 | 13 |
| Pancreatic Adenocarcinoma Signaling | 1.38038E-08 | 0% | Activated | 2.828 | 22 |
| ERK/MAPK Signaling | 8.12831E-08 | 0% | Activated | 2.268 | 29 |
| Systemic Lupus Erythematosus In B Cell Signaling Pathway | 8.91251E-08 | 0% | Activated | 4.333 | 36 |
| IL-15 Signaling | 1.02329E-07 | 0% | Activated | 2.668 | 17 |
| Leukocyte Extravasation Signaling | 1.28825E-07 | 0% | Activated | 2.746 | 29 |
| IL-2 Signaling | 1.8197E-07 | 0% | Activated | 3.357 | 15 |
| GM-CSF Signaling | 2.13796E-07 | 0% | Activated | 2.84 | 16 |
| IL-3 Signaling | 2.29087E-07 | 0% | Activated | 3.153 | 17 |
| Crosstalk between Dendritic Cells and Natural Killer Cells | 2.75423E-07 | 0% | Activated | 3.742 | 18 |
| Thrombopoietin Signaling | 2.88403E-07 | 0% | Activated | 2.324 | 15 |
| CD28 Signaling in T Helper Cells | 3.80189E-07 | 0% | Activated | 3.638 | 21 |
| PI3K/AKT Signaling | 4.67735E-07 | 0% | Activated | 2.138 | 26 |
| Tec Kinase Signaling | 4.7863E-07 | 0% | Activated | 2.294 | 25 |
| Oncostatin M Signaling | 7.07946E-07 | 0% | Activated | 3.317 | 12 |
| PDGF Signaling | 8.12831E-07 | 0% | Activated | 2.183 | 17 |
| JAK/Stat Signaling | 1.44544E-06 | 0% | Activated | 3.5 | 16 |
| NF-κB Activation by Viruses | 2.04174E-06 | 0% | Activated | 2.5 | 16 |
| fMLP Signaling in Neutrophils | 3.71535E-06 | 0% | Activated | 2.524 | 19 |
| Acute Myeloid Leukemia Signaling | 6.30957E-06 | 0% | Activated | 2.496 | 16 |
| iCOS-iCOSL Signaling in T Helper Cells | 7.76247E-06 | 0% | Activated | 3.5 | 18 |
| Cardiac Hypertrophy Signaling (Enhanced) | 8.70964E-06 | 0% | Activated | 3.202 | 47 |
| FAT10 Cancer Signaling Pathway | 1.07152E-05 | 0% | Activated | 2.714 | 11 |
| Natural Killer Cell Signaling | 1.38038E-05 | 0% | Activated | 2.2 | 25 |
| Senescence Pathway | 1.54882E-05 | 0% | Activated | 3.413 | 31 |
| Renin-Angiotensin Signaling | 1.8197E-05 | 0% | Activated | 2.5 | 18 |
| Integrin Signaling | 1.86209E-05 | 0% | Activated | 3.128 | 26 |
| Colorectal Cancer Metastasis Signaling | 2.13796E-05 | 0% | Activated | 2.041 | 29 |
| PKCθ Signaling in T Lymphocytes | 2.45471E-05 | 0% | Activated | 4.025 | 21 |
| Estrogen-mediated S-phase Entry | 2.34423E-05 | 0% | Inhibited | -2.121 | 8 |
| Myc Mediated Apoptosis Signaling | 2.45471E-05 | 0% | Inhibited | -2.111 | 11 |
| VEGF Signaling | 2.51189E-05 | 0% | Activated | 2.324 | 16 |
| Th1 Pathway | 2.5704E-05 | 0% | Activated | 3 | 18 |
| Phospholipase C Signaling | 2.81838E-05 | 0% | Activated | 2.6 | 29 |
| HGF Signaling | 2.95121E-05 | 0% | Activated | 2.138 | 17 |
| Type I Diabetes Mellitus Signaling | 2.95121E-05 | 0% | Activated | 2.121 | 17 |
| Growth Hormone Signaling | 3.71535E-05 | 0% | Activated | 2.309 | 13 |
| B Cell Receptor Signaling | 4.16869E-05 | 0% | Activated | 4.146 | 23 |
| LPS-stimulated MAPK Signaling | 4.2658E-05 | 0% | Activated | 2.138 | 14 |
| Lymphotoxin β Receptor Signaling | 4.36516E-05 | 0% | Activated | 2.121 | 11 |
| Estrogen-Dependent Breast Cancer Signaling | 5.88844E-05 | 0% | Activated | 3.051 | 13 |
| Angiopoietin Signaling | 6.76083E-05 | 0% | Activated | 2.111 | 13 |
| ErbB2-ErbB3 Signaling | 6.76083E-05 | 0% | Activated | 2.887 | 12 |
| Role of NFAT in Regulation of the Immune Response | 8.51138E-05 | 0% | Activated | 3.578 | 22 |
| Apoptosis Signaling | 9.54993E-05 | 0% | Inhibited | -2.324 | 15 |
| Dendritic Cell Maturation | 0.0001 | 0% | Activated | 3.578 | 22 |
| Purine Nucleotides De Novo Biosynthesis II | 0.000102329 | 0% | Inhibited | -2.236 | 5 |
| RAN Signaling | 0.000107152 | 0% | Inhibited | -2.449 | 6 |
| Apelin Endothelial Signaling Pathway | 0.000162181 | 0% | Activated | 2.5 | 16 |
| PPARα/RXRα Activation | 0.00017378 | 0% | Inhibited | -2.236 | 22 |
| Actin Cytoskeleton Signaling | 0.000194984 | 0% | Activated | 3.578 | 24 |
| NF-κB Signaling | 0.000204174 | 0% | Activated | 2.4 | 21 |
| Neuregulin Signaling | 0.000245471 | 0% | Activated | 2.138 | 14 |
| iNOS Signaling | 0.000288403 | 0% | Activated | 2.828 | 9 |
| CD40 Signaling | 0.000301995 | 0% | Activated | 2.111 | 11 |
| Cell Cycle Control of Chromosomal Replication | 0.000363078 | 0% | Inhibited | -3.162 | 10 |
| Production of Nitric Oxide and Reactive Oxygen Species in Macrophages | 0.000398107 | 0% | Activated | 2.828 | 21 |
| PTEN Signaling | 0.000467735 | 0% | Inhibited | -2.5 | 16 |
| Mouse Embryonic Stem Cell Pluripotency | 0.000512861 | 0% | Activated | 2.496 | 14 |
| Pyrimidine Deoxyribonucleotides De Novo Biosynthesis I | 0.000524807 | 0% | Inhibited | -2.449 | 6 |
| Cdc42 Signaling | 0.000588844 | 0% | Activated | 2.53 | 19 |
| TGF-β Signaling | 0.000831764 | 0% | Activated | 3.051 | 13 |
| IL-22 Signaling | 0.000870964 | 0% | Activated | 2.449 | 6 |
| Neuroinflammation Signaling Pathway | 0.000912011 | 0% | Activated | 3.266 | 28 |
| Signaling by Rho Family GTPases | 0.001 | 0% | Activated | 2.711 | 24 |
| RhoA Signaling | 0.001047129 | 0% | Activated | 2.138 | 15 |
| Rac Signaling | 0.001202264 | 0% | Activated | 3.207 | 14 |
| PI3K Signaling in B Lymphocytes | 0.001258925 | 0% | Activated | 3.873 | 16 |
| HMGB1 Signaling | 0.001318257 | 0% | Activated | 2.496 | 18 |
| p70S6K Signaling | 0.001698244 | 1% | Activated | 2.496 | 15 |
| FLT3 Signaling in Hematopoietic Progenitor Cells | 0.001819701 | 1% | Activated | 3.317 | 11 |
| Gα12/13 Signaling | 0.001819701 | 1% | Activated | 3.207 | 15 |
| PPAR Signaling | 0.001778279 | 1% | Inhibited | -2.496 | 13 |
| ErbB Signaling | 0.002187762 | 1% | Activated | 2.309 | 12 |
| Actin Nucleation by ARP-WASP Complex | 0.002630268 | 1% | Activated | 2.121 | 10 |
| HIPPO signaling | 0.002951209 | 1% | Activated | 2.236 | 11 |
| B Cell Activating Factor Signaling | 0.003548134 | 1% | Activated | 2.236 | 7 |
| CD27 Signaling in Lymphocytes | 0.004168694 | 1% | Activated | 2.646 | 8 |
| IL-17A Signaling in Airway Cells | 0.004365158 | 1% | Activated | 2.333 | 9 |
| CNTF Signaling | 0.006456542 | 2% | Activated | 2.828 | 8 |
| Osteoarthritis Pathway | 0.008317638 | 2% | Activated | 2.324 | 19 |
| IL-6 Signaling | 0.008709636 | 2% | Activated | 3.606 | 13 |
| RANK Signaling in Osteoclasts | 0.011220185 | 3% | Activated | 3 | 10 |
| tRNA Charging | 0.011220185 | 3% | Inhibited | -2.449 | 6 |
| NER Pathway | 0.012302688 | 3% | Inhibited | -3.317 | 11 |
| Paxillin Signaling | 0.017378008 | 4% | Activated | 2.828 | 11 |
| BMP signaling pathway | 0.023442288 | 5% | Activated | 2.333 | 9 |
| NGF Signaling | 0.024547089 | 5% | Activated | 3.162 | 11 |
|  |  |  |  |  |  |
| **(–)-Hopeaphenol vs. No treatment** | | | | | |
| **Signaling pathway** | **p** | **FDR** | **State** | **Z** | **N genes** |
| IL-15 Signaling | 0.00047863 | 5% | Inhibited | -2.84 | 15 |
|  |  |  |  |  |  |
| **PMA + (–)-Hopeaphenol vs. PMA** | | | | | |
| **Signaling pathway** | **p** | **FDR** | **State** | **Z** | **N genes** |
| IL-15 Signaling | 6.60693E-05 | 1% | Inhibited | -3.153 | 17 |
| Small Cell Lung Cancer Signaling | 0.000114815 | 1% | Inhibited | -2.496 | 16 |
| PD-1, PD-L1 cancer immunotherapy pathway | 0.000660693 | 2% | Activated | 2.5 | 19 |
| Tec Kinase Signaling | 0.000562341 | 2% | Inhibited | -2.4 | 27 |
| IL-2 Signaling | 0.000870964 | 2% | Inhibited | -2.309 | 13 |
| Reelin Signaling in Neurons | 0.001513561 | 3% | Inhibited | -2.065 | 20 |
| Gαq Signaling | 0.00162181 | 3% | Inhibited | -2.837 | 24 |
| Non-Small Cell Lung Cancer Signaling | 0.001659587 | 3% | Inhibited | -2.111 | 14 |
| PI3K/AKT Signaling | 0.003235937 | 3% | Inhibited | -2.828 | 26 |
| Systemic Lupus Erythematosus In B Cell Signaling Pathway | 0.003981072 | 4% | Inhibited | -3.212 | 35 |
| ErbB2-ErbB3 Signaling | 0.004786301 | 4% | Inhibited | -2.111 | 12 |
| Dermatan Sulfate Biosynthesis | 0.006309573 | 5% | Inhibited | -2.111 | 11 |
